# Development of microsatellite markers for a Chinese endemic plant, *Torreya yunnanensis* (Taxaceae)

**DOI:** 10.1002/aps3.1145

**Published:** 2018-05-10

**Authors:** Yu Peng Wang, Hai Xiang Cheng, Jian Hui Li, Shou Biao Zhou

**Affiliations:** ^1^ College of Life Sciences Anhui Normal University Wuhu Anhui Province People's Republic of China; ^2^ School of Resources and Environment Anhui Agricultural University Hefei Anhui Province People's Republic of China; ^3^ Quzhou Academy of Agricultural Sciences Quzhou Zhejiang Province 324000 People's Republic of China

**Keywords:** genetic diversity, microsatellite, simple sequence repeat (SSR) marker, Taxaceae, *Torreya yunnanensis*

## Abstract

**Premise of the Study:**

Microsatellite markers were developed in *Torreya yunnanensis* (Taxaceae) to investigate the genetic diversity, phylogeography, and population structure of the species.

**Methods and Results:**

Sixteen primer sets were identified using Illumina 2 × 100‐bp paired‐end sequencing and bioinformatic screening. Most primers also amplified in *T. fargesii*,* T*. *grandis*,* T. jackii*, and *T. nucifera*.

**Conclusions:**

These results indicate the utility of these microsatellite markers in *T. yunnanensis* for future studies of molecular ecology as well as their applicability across the genus.

*Torreya yunnanensis* C. Y. Cheng & L. K. Fu (Taxaceae) is a Chinese endemic evergreen tree that is highly valued for its timber, as well as for its ornamental and ecological benefits. Wild resources of *T. yunnanensis* have declined sharply as a result of overexploitation and deforestation. It was listed as endangered in the *China Species Red List* (Wang and Xie, 2004) and is now rare in the field. Very little research has been done on this species, except for recent studies on chemical composition, cultivation techniques, and community structure (e.g., Hou et al., 2015; Fu et al., 2016; Li et al., 2016). Although conservation of this species is urgent, little is known about its genetic diversity, which is important for planning conservation strategy (Avise and Hamrick, 1996; Jiang et al., 1997).

Microsatellites, also called simple sequence repeat (SSR) markers, are a neutral molecular marker widely distributed in the nuclear genome of eukaryotes. Because of advantages such as high polymorphism, ability to facilitate genotyping, and low demand of DNA quality, microsatellites have been widely used as DNA markers in population genetic studies and parentage analysis. In this study, 16 polymorphic microsatellite markers were developed using Illumina 2 × 100‐bp paired‐end sequencing and bioinformatics screening. We believe these markers are useful for investigating genetic diversity and population structure for *T. yunnanensis* and other *Torreya* species.

## METHODS AND RESULTS

A total of 64 *T. yunnanensis* individuals were sampled from five wild populations in this study (Appendix 1). The sample sizes were small for each population (ranged from 7–18) because *T. yunnanensis* is an endangered species and is rare in the field. Young leaves were collected and dry preserved with silica gel. Genomic DNA was extracted using the cetyltrimethylammonium bromide (CTAB) method (Doyle, 1987). A normalized DNA library was constructed using the TruSeq Stranded DNA Sample Preparation kit (Illumina Inc., San Diego, California, USA) for one sample from the Misha population. The normalized DNA library was sequenced using the Illumina HiSeq 2500 (Illumina Inc.). A total of 5,362,980,012‐bp paired‐end raw sequences were trimmed to remove adapter sequences and low‐quality sequences using Trim Galore (http://www.bioinformatics.babraham.ac.uk/projects/trim_galore/). De novo assembly based on trim reads was performed with Velvet 1.2.10 (Zerbino and Birney, 2008). A total of 50,381,156 trim reads with an average length of 98.7 bp (4,975,006,766 bp in total) were produced and de novo assembled into 283,057 contigs with an N50 length of 1823 bp. SSR detection was performed with MISA (Thiel et al., 2003) using the following criteria: ≥6 repeat units for dinucleotides, ≥5 for tri‐, and ≥4 for tetra‐, penta‐, and hexanucleotides. A total of 4146 SSRs were identified. Of them, trinucleotide repeat motifs (50.19%) were the most common, followed by di‐ (44.19%), tetra‐ (3.14%), penta‐ (0.58%), and hexanucleotide (1.91%) repeat motifs. Primer pairs were designed using Primer3 (Koressaar and Remm, 2007; Untergasser et al., 2012) with the default conditions.

Fifty primer pairs amplifying SSRs containing dinucleotide or trinucleotide motifs were randomly selected and tested in four individuals. Sixteen primer sets displaying consistent amplification (Table 1) were selected for further polymorphism tests on 64 individuals. All PCRs were performed in 20‐μL volume (50–100 ng of genomic DNA, 10 μL of 2× EasyTaq PCR SuperMix polymerase [TransGen Biotech Co., Beijing, China], 0.5 μM of each primer pair [the forward primer was fluorescently labeled with FAM, HEX, or TAMRA]) under the following conditions: 5 min denaturation at 95°C; 32 cycles of 30 s at 95°C, 1 min at specific annealing temperatures, and 1 min at 72°C (Table 1); and a final extension of 72°C for 10 min. PCR products were separated on an ABI PRISM 3730 Genetic Analyzer (Thermo Fisher Scientific, Waltham, Massachusetts, USA) with a GeneScan 500 LIZ Size Standard. Allele peaks were scored using GeneMarker (version 1.3; SoftGenetics, State College, Pennsylvania, USA). Allele size range, number of alleles (*A*), observed heterozygosity (*H*_o_), expected heterozygosity (*H*_e_), and fixation index (*F*) of each locus for each population were calculated using GENETIX version 4.0 (Belkhir et al., 2001). MICRO‐CHECKER 2.2.3 (van Oosterhout et al., 2004) was used to detect genotyping errors due to null alleles, stuttering, or allele dropout. Deviation from Hardy–Weinberg equilibrium was tested with GENEPOP version 3.4 (Rousset, 2008), and the *P* values were then tested using Bonferroni correction. The exact tests for genotypic linkage disequilibrium for each pair of loci in each population were performed with GENEPOP version 3.4.

**Table 1:** Characterization of 16 *Torreya yunnanensis* microsatellite loci

Locus	Primer sequences (5′–3′)	Repeat motif	Allele size range (bp)	*A*	*T*_a_ (°C)	GenBank accession no.
Ty12662	F: CAGCGCACATTAAATCGGTA	(TA)_9_	161–201	12	48	KX359344
	R: CTTCAACCCCGACTCTGCTA					
Ty13451	F: GCTTGCTTGGAAACTTTACCC	(GA)_9_	203–221	6	56	KX359348
	R: GCATGCCCAGTTCTCAGTTA					
Ty13986	F: TGTGCAGCACCTAAACAGAGA	(AG)_9_	177–245	4	56	KX359346
	R: TCATCTGCTTTGTGCCTCTTT					
Ty23324	F: CTTGACAGGACATTACCAGGA	(AT)_9_	143–173	9	52	KX359341
	R: CGTGGGATCCCTTGCTTAT					
Ty39589	F: GCCATGCGATGGTATTCTTC	(TTC)_7_	173–182	4	52	KX359339
	R: CCTGTGGCATACTTGCTGTG					
Ty41047	F: TCGCTGAGAGCCTATCTGGT	(AT)_7_	159–199	14	54	KX359350
	R: CAAAAGCGTTCTGCAAACAA					
Ty46473	F: TGTGGATCAAGGAATGTGGA	(ATT)_7_	203–251	4	51	KX359349
	R: TGTGATGGTCTCCTCCATGA					
Ty49984	F: AGAGGGTCTTGATGGGGACT	(TA)_9_	179	1	55	KX359342
	R: TGGAACAATACCACTGCTGAA					
Ty53899	F: CCATGGCCAGCTTCAATTAT	(GAA)_6_	173–215	5	48	KX359340
	R: CATGTTGAGCACCCTGATTG					
Ty60665	F: TTGGCTGCAATATGACAAGA	(AT)_8_	179–249	10	52	KX359343
	R: TGCAGGGCATATGTACAAAAA					
Ty61395	F: TGTGGAAAGGTGGTGAACAA	(GA)_9_	189	1	53	KX359345
	R: CCAATTTGTGGAGCGTTTCT					
Ty72305	F: CAGATGCAAGAAAGGAACCA	(AT)_9_	125–173	5	50	KX359336
	R: ATTGGCAAATATGCCCTTTT					
Ty74507	F: GCAGATTGGGAGGCATTTT	(AT)_8_	107–167	6	49	KX359347
	R: AACGCGTTTTGGTTCATTTC					
Ty78313	F: AGCTAGAGCCAAATACACAGAA	(ATA)_6_	187–193	2	52	KX359337
	R: GGGTGGTTAGAACCTCTCACG					
Ty79697	F: TTGGAGATTCATGGGGAGAG	(GA)_8_	215–307	18	52	KX359351
	R: GGGTTGTGATGCTCTTGGAT					
Ty80072	F: AAAGCTACAGCTGTGTTTGTCA	(AT)_9_	145–197	10	51	KX359338
	R: TGCTAAAGCTCAACGCCATA					

Note

*A* = number of alleles; *T*_a_ = annealing temperature.

According to MICRO‐CHECKER analysis, there is no evidence for scoring error due to stuttering, large allele dropout, or the existence of null alleles. Fourteen loci were polymorphic in five wild populations (Table 2) and two loci (Ty49984 and Ty61395) were monomorphic. Values for *A*,* H*_o_, and *H*_e_ of 14 loci ranged from 2–12, 0.000–1.000, and 0.000–0.869 (Table 2), respectively, in five populations. The *H*_o_ and *H*_e_ for all five populations, calculated from data from the 14 loci, ranged from 0.664–0.728 and 0.514–0.633 (data not shown); these values were higher than those found in *T. jackii* Chun (*H*_o_ = 0.5012, *H*_e_ = 0.4830; Li, 2013), *Taxus chinensis* (Pilg.) Rehder (*H*_o_ = 0.107, *H*_e_ = 0.121; Vu et al., 2017), and *Taxus cuspidata* Siebold & Zucc. (*H*_o_ = 0.263, *H*_e_ = 0.028; Cheng et al., 2015). Significant deviation from Hardy–Weinberg equilibrium was found in loci Ty13986, Ty53899, Ty60665, and Ty72305 (*P* < 0.05) after Bonferroni correction. Most of the *F* values were negative, indicating low levels of inbreeding in these populations. There was no evidence of linkage disequilibrium among pairs of loci in the sample. The sequences of these microsatellite loci have been deposited in the GenBank database (Table 1), and the raw sequences from high‐throughput sequencing have been deposited in the Sequence Read Archive (SRA) of the National Center for Biotechnology Information (NCBI; accession no. SRR6428886).

**Table 2:** Genetic diversity of 14 polymorphic SSR markers developed in *Torreya yunnanensis*.^a^

Locus	Misha (*N* = 7)				Xiangtu (*N* = 10)				Baijixun (*N* = 12)				Baohe (*N* = 17)				Weideng (*N* = 18)			
	*A*	*H*_o_	*H*_e_ b	*F*	*A*	*H*_o_	*H*_e_ b	*F*	*A*	*H*_o_	*H*_e_ b	*F*	*A*	*H*_o_	*H*_e_ b	*F*	*A*	*H*_o_	*H*_e_ b	*F*
Ty12662	4	0.800	0.580	−0.379	6	1.000	0.720	−0.379	7	1.000	0.788	−0.379	11	1.000	0.869	−0.379	11	1.000	0.818	−0.379
Ty13451	2	0.571	0.408	−0.400	3	0.600	0.445	−0.400	4	0.750	0.608	−0.400	5	0.588	0.633	−0.400	5	0.722	0.701	−0.400
Ty13986	3	0.800	0.540	−0.481	2	0.700	0.455	−0.481	3	0.455	0.541*	−0.481	3	0.500	0.538	−0.481	3	0.750	0.569	−0.481
Ty23324	4	1.000	0.684	−0.463	5	0.700	0.735	−0.463	8	1.000	0.833	−0.463	6	0.941	0.763	−0.463	7	0.944	0.824	−0.463
Ty39589	2	0.143	0.133	−0.077	2	0.100	0.095	−0.077	3	0.417	0.622	−0.077	3	0.471	0.486	−0.077	3	0.667	0.586	−0.077
Ty41047	3	1.000	0.622	−0.607	5	1.000	0.770	−0.607	6	0.917	0.726	−0.607	10	1.000	0.791	−0.607	9	1.000	0.826	−0.607
Ty46473	2	1.000	0.500	−1.000	2	1.000	0.500	−1.000	2	1.000	0.500	−1.000	4	0.929	0.666	−1.000	2	1.000	0.500	−1.000
Ty53899	2	0.143	0.500	0.714	2	0.111	0.105	0.714	4	0.364	0.442	0.714	4	0.143	0.403**	0.714	3	0.167	0.156	0.714
Ty60665	3	0.571	0.500	−0.143	5	0.667	0.654	−0.143	8	0.667	0.826	−0.143	8	0.688	0.811	−0.143	6	0.235	0.787***	−0.143
Ty72305	1	0.000	0.000	NA	1	0.000	0.000	NA	4	0.167	0.413*	NA	3	0.059	0.112*	NA	4	0.167	0.252	NA
Ty74507	4	1.000	0.704	−0.420	3	0.800	0.660	−0.420	6	0.833	0.684	−0.420	4	0.529	0.500	−0.420	4	0.833	0.582	−0.420
Ty78313	2	1.000	0.500	−1.000	2	1.000	0.500	−1.000	2	1.000	0.500	−1.000	2	1.000	0.500	−1.000	2	1.000	0.500	−1.000
Ty79697	7	0.857	0.796	−0.077	6	1.000	0.785	−0.077	8	0.917	0.701	−0.077	12	0.800	0.724	−0.077	12	0.824	0.690	−0.077
Ty80072	4	1.000	0.735	−0.361	6	0.900	0.770	−0.361	6	0.833	0.681	−0.361	8	0.647	0.592	−0.361	9	0.889	0.696	−0.361

Note

*A* = number of alleles; *F* = fixation index; *H*_e_ = expected heterozygosity; *H*_o_ = observed heterozygosity; *N* = sample size; NA = not applicable.

a Locality and voucher information are provided in Appendix 1.

^b^Significant deviations from Hardy–Weinberg equilibrium at *P < 0.05, **P < 0.01, ***P < 0.001 after Bonferroni correction.

Cross‐amplification of these 16 primer sets was tested in five individuals in each of the following species: *T. fargesii* Franch., *T. grandis* Fortune ex Lindl., *T. jackii*, and *T. nucifera* (L.) Siebold & Zucc. Most primers also amplified in these species (Table 3).

**Table 3:** Results of cross‐amplification (allele size ranges) for the 16 microsatellite markers developed for *Torreya yunnanensis* in four related *Torreya* species.^a^

Locus	*T. fargesii* (*N* = 5)	*T*. *grandis* (*N* = 5)	*T. nucifera* (*N* = 5)	*T. jackii* (*N* = 5)
Ty12662	165–171	175–191	205–209	185
Ty13451	—	215–223	203–207	195–205
Ty13986	210–225	—	195–205	—
Ty23324	135–149	155–163	169	145–161
Ty39589	173–176	179–185	170–173	170
Ty41047	165	173–181	177–179	189–193
Ty46473	206–212	248	215–230	224–233
Ty49984	179	179	179	179
Ty53899	—	—	—	179–182
Ty60665	189–215	195–199	175–193	225–229
Ty61395	189	189	189–191	—
Ty72305	150–159	185–187	135–147	163–169
Ty74507	105–121	—	127–143	169–171
Ty78313	—	190–193	—	193
Ty79697	275–297	257–259	231–245	289–295
Ty80072	135–145	163–189	147–149	159–163

Note

— = failed amplification.

a Locality and voucher information are provided in Appendix 1.

## CONCLUSIONS

Genetic diversity in wild *T. yunnanensis* populations was shown to be high. The SSR markers that we developed were polymorphic and will be a useful tool to investigate the genetic diversity, population structure, and levels of gene flow, as well as to optimize breeding for *T. yunnanensis* and related species.

## Appendix 1 Voucher information for *Torreya* species used in this study.

**Table:**

Species	Voucher ID^a^,^b^	*N*	Collection locality	Geographic coordinates
*T. yunnanensis* C. Y. Cheng & L. K. Fu	Ty‐001‐XH	7	Misha, Yunnan, China	26°15′57″N, 99°38′08″E
*T. yunnanensis*	Ty‐002‐XH	10	Xiangtu, Yunnan, China	26°15′22″N, 99°34′20″E
*T. yunnanensis*	Ty‐003‐XH	12	Baijixun, Yunnan, China	27°23′50″N, 99°01′24″E
*T. yunnanensis*	Ty‐004‐XH	17	Baohe, Yunnan, China	27°03′24″N, 99°20′37″E
*T. yunnanensis*	Ty‐005‐XH	18	Weideng, Yunnan, China	27°07′50″N, 99°15′7″E
*T. fargesii* Franch.	Tf‐001‐XH	5	Fangxian, Hubei, China	31°49′57″N, 110°37′46″E
*T*. *grandis* Fortune ex Lindl.	Tg‐001‐XH	5	Zhuji, Zhejiang, China	29°42′28″N, 120°05′51″E
*T. jackii* Chun	Tj‐001‐XH	5	Xianju, Zhejiang, China	28°46′32″N, 120°47′12″E
*T. nucifera* (L.) Siebold & Zucc.	Tn‐001‐XH	5	Hangzhou, Zhejiang, China	30°06′56″N, 120°00′02″E

NOTES

*N* = sample size.

a Vouchers deposited at Anhui Normal University. The voucher ID for each sample is sufficient to identify the exact voucher of these samples.

b XH in the voucher ID identifies the collector, Xin Hong.
